# Creating opportunities through mentorship, parental involvement, and safe spaces (COMPASS) program: multi-country study protocol to protect girls from violence in humanitarian settings

**DOI:** 10.1186/s12889-016-2894-3

**Published:** 2016-03-05

**Authors:** Kathryn L. Falb, Sophie Tanner, Leora Ward, Dorcas Erksine, Eva Noble, Asham Assazenew, Theresita Bakomere, Elizabeth Graybill, Carmen Lowry, Pamela Mallinga, Amy Neiman, Catherine Poulton, Katie Robinette, Marni Sommer, Lindsay Stark

**Affiliations:** International Rescue Committee, 122 E 42nd St, New York City, NY 10168 USA; International Rescue Committee, 3 Bloomsbury Place, London, WC1A 2QL UK; International Rescue Committee, 1730 M Street, NW - Suite 505, Washington DC, 20036 USA; International Rescue Committee, TK International Bldg. 6th Floor, Bole Rd, Addis Ababa, Ethiopia; International Rescue Committee, Bukavu, Democratic Republic of Congo; International Rescue Committee, Nairobi i-HUB, Galana Plaza, Wing B, 4th Floor, Kilimani, Nairobi, Kenya; International Rescue Committee, Place de la Vieille Halle aux Blés 16, 1000 Bruxelles, Belgium; Columbia University Mailman School of Public Health, 722 W. 168th Street, Room 537, New York, NY 10032 USA; Columbia University Mailman School of Public Health, 60 Haven Avenue, B-4, Suite 432, New York, NY 10032 USA

**Keywords:** Sexual violence, Child abuse and neglect, Violence against women and girls, Behavior change, Randomized controlled trial, Evaluation, Democratic Republic of Congo, Ethiopia

## Abstract

**Background:**

Violence against adolescent girls in humanitarian settings is of urgent concern given their additional vulnerabilities to violence and unique health and well-being needs that have largely been overlooked by the humanitarian community. In order to understand what works to prevent violence against adolescent girls, a multi-component curriculum-based safe spaces program (Creating Opportunities through Mentorship, Parental involvement and Safe Spaces – COMPASS) will be implemented and evaluated. The objectives of this multi-country study are to understand the feasibility, acceptability and effectiveness of COMPASS programming to prevent violence against adolescent girls in diverse humanitarian settings.

**Methods/design:**

Two wait-listed cluster-randomized controlled trials are being implemented in conflict-affected communities in eastern Democratic Republic of Congo (N = 886 girls aged 10–14 years) and in refugee camps in western Ethiopia (N = 919 girls aged 13–19 years). The intervention consists of structured facilitated sessions delivered in safe spaces by young female mentors, caregiver discussion groups, capacity-building activities with service providers, and community engagement. In Ethiopia, the research centers on the overall impact of COMPASS compared to a wait-list group. In DRC, the research objective is to understand the incremental effectiveness of the caregiver component in addition to the other COMPASS activities as compared to a wait-list group. The primary outcome is change in sexual violence. Secondary outcomes include decreased physical and emotional abuse, reduced early marriage, improved gender norms, and positive interpersonal relationships, among others. Qualitative methodologies seek to understand girls’ perceptions of safety within their communities, key challenges they face, and to identify potential pathways of change.

**Discussion:**

These trials will add much needed evidence for the humanitarian community to meet the unique needs of adolescent girls and to promote their safety and well-being, as well as contributing to how multi-component empowerment programming for adolescent girls could be adapted across humanitarian settings.

**Trial registration:**

Clinical Trials NCT02384642 (Registered: 2/24/15) & NCT02506543 (Registered: 7/19/15).

## Background

Globally, approximately 59.5 million people were forcibly displaced in 2014 [1], which is one of the highest levels seen in decades. Throughout periods of armed conflict and displacement, women and girls face increased risk of violence due to a constellation of factors that may increase their vulnerabilities, such as weakened community protection mechanisms, existing inequitable and evolving gender norms, financial instability, limited economic opportunities, and separation from family members [2–4]. In fact, approximately one in five displaced women report experiencing sexual violence [5]. Intimate partner violence (IPV) and other forms of abuse or exploitation may also occur at higher frequencies and are of key concerns for conflict-affected women and girls [6, 7].

Within humanitarian emergencies, female adolescents aged 10–19 years are particularly at risk of sexual violence, sexual exploitation, and early marriage [2, 3, 8]. For example, the percentage of registered marriages which involved girls below age 18 doubled during two years of the Syrian conflict. This is thought to be due to a complex set of circumstances that families face in displacement which lead them to see marriage as the best option for gaining resources and supplies as well as protecting their daughters from violence outside the home [9, 10]. Adolescent pregnancy is also of concern for child brides within early and forced marriages. These young females may be expected and pressured to become pregnant as the result of inequitable gender norms and power dynamics, or due to the lack of access to contraceptive methods in emergencies, or pregnancy as the direct result of forced sex. [11, 12].

Not only do adolescent girls face increased vulnerability to violence, similar to development contexts, the needs of adolescent girls are often overlooked by communities, families, and service providers during and after conflict due to their lack of social status, stemming from gender inequalities and the invisibility of younger populations. Girls may also have limited mobility and therefore, face restricted access to pertinent life-saving information, schooling, and health and protection services [13, 14]. Limited understanding and investment to date by the humanitarian community of the specific factors that shape adolescent girls’ vulnerabilities and needs has led to inadequate resources and programming for this unique population [4, 15, 16].

Despite the multitude of reports on violence against women and girls in humanitarian settings and the damaging physical and mental health effects resulting from such violence, few rigorous studies have examined how best to prevent violence in such contexts [17, 18]. In fact, only two prevention studies focusing on reducing IPV in humanitarian settings have been completed to date, both of which only focused on adult women [19, 20]. Simultaneously, efforts in child protection research have demonstrated a small, but expanding evidence base for the use of safe spaces to support children’s psychosocial wellbeing in emergencies. The goals of such safe spaces, which can generally be constructed quickly in an emergency, are to provide children with a place to participate in activities and receive support to promote their overall well-being and resilience [21]. However, it remains unclear if safe space programming confers additional protection against the unique risks of violence that adolescent girls face [22, 23], particularly as most child friendly spaces do not have tailored programming by gender. Ultimately, while there are advances in IPV prevention strategies and child protection programming [19, 20], there is a glaring gap of rigorous evidence on how best to reduce violence against adolescent girls in emergencies.

To address this gap in the evidence, Columbia University and the International Rescue Committee (IRC), are undertaking an evaluation of the Creating Opportunities through Mentoring, Parental involvement, and Safe Spaces (COMPASS) program, of which a key strategy is to develop the skills and assets of girls with the support of female mentors in ‘safe spaces’, alongside additional engagement of caregivers and capacity-building of service providers. The objectives of this multi-country study are to understand the feasibility, acceptability and effectiveness of these approaches to respond to and prevent violence against adolescent girls in three diverse humanitarian settings.

## Methods

### Study design

Over three years (2014–2017), the COMPASS program is being implemented and evaluated in conflict-affected communities in the Democratic Republic of Congo (DRC), Sudanese refugee camps in Western Ethiopia, and in sites across Khyber Pakhtunkwa province in Pakistan, including Jalozai camp. In DRC and Ethiopia, the study design employs a wait-listed two-arm cluster randomized controlled trial. Given the exceedingly restrictive environment in Pakistan around issues of violence against girls, a mixed methods pre-post test is being implemented rather than an impact evaluation. Thus, the remainder of the study protocol manuscript will center on the trials taking place in DRC and Ethiopia. Further details of all study designs are found in Table 1. Qualitative inquiry will be used to complement the quantitative findings and to provide more nuanced understanding of processes of change and acceptability of programming.

**Table 1:** Key study characteristics by COMPASS country site

Site	Primary research question	Study design	Quantitative surveys	Quantitative sample size at baseline
Conflict-affected communities in Kabare and Uvira territories, South Kivu Province, Democratic Republic of Congo	What is the incremental effectiveness of the parental curriculum, in addition to the core COMPASS programming, on improving girls’ safety and well-being?	Wait-list cluster randomized controlled trial + qualitative	10-12 year old CAPI survey^a^	886 girls (10–14 years)
			13-14 year old CAPI + ACASI survey	777 primary caregivers for girls
			Caregiver survey	
Refugee camps (Sherkole, Tongo, and Bambasi), Benishangul-Gumuz Region, Ethiopia	What is the overall effectiveness of the COMPASS program on improving girls’ safety and well-being, as compared to girls in the wait-list arm?	Wait-list cluster randomized controlled trial + qualitative	13-19 year old ACASI survey	919 girls (13–19 years)
Communities, and Jalozai camp in Khyber Paktunkwah Province, Pakistan	What is the overall acceptability and feasibility of COMPASS programming?	Pre-post test + qualitative	12-19 year old paper-based survey	208 girls (12–19 years)

^a^The 10–12 year old survey does not contain sensitive questions of sexual health

### Study settings

Eastern DRC has grappled with protracted conflict for decades, vacillating between periods of relative calm and instability. North and South Kivu provinces in eastern DRC are home to approximately 60 % of DRC’s 2.6 million internally displaced persons as well as continual influxes of refugees from Burundi, Rwanda, or other neighboring countries [24]. Violence against women in eastern DRC is widespread, as up to 40 % of women report lifetime experience of sexual violence [25, 26]. While less is known about the experiences of adolescent girls in eastern DRC, non-representative monitoring data from IRC partners documented an increase from 12 % to 28 % of girls aged 12–17 years among their caseloads of survivors between January and September 2012 during outbreaks of violence. At study inception, COMPASS was planned to take place in both North and South Kivu, yet, given unpredictable outbreaks of violence and security concerns for program and research staff in North Kivu, the study setting was refined to take place in fourteen communities in Kabare and Uvira territories, South Kivu. IRC has been operating in eastern DRC since 1996 and has been running a large and comprehensive program to protect and empower women and girls. This program includes health care and case management for survivors, capacity-building activities with community-based organizations, socio-economic activities and primary prevention interventions, in addition to general emergency response programming.

Three refugee camps in the Benishangul-Gumuz region of Ethiopia, bordering Sudan and South Sudan, serve as the second study site. Approximately 38,000 people reside in these three primarily Sudanese refugee camps (Sherkole, Tongo, and Bambasi), which are characterized by multiple safety and protection concerns. Documented in the IRC Community Wellbeing Initiative (CWI) programming assessments, concerns include high risks of adolescent girls experiencing sexual violence prior to arrival in Ethiopia and early or forced marriage. Often, these marriages are seen as a mechanism to ‘protect’ girls from sexual violence or as a means for girls’ to meet their basic needs [27]. These camps, established in 2011–2012, are highly diverse with numerous ethnicities of refugees and languages spoken. IRC has been operating in these camps since 2014 and provides community well-being programming, health programming, and water/ sanitation services, amongst other programs. Specific CWI programming includes providing access to quality health, psychosocial, and legal protections for survivors of gender-based violence, and community mobilization activities across the refugee camps.

### Intervention description

**Fig 1:**
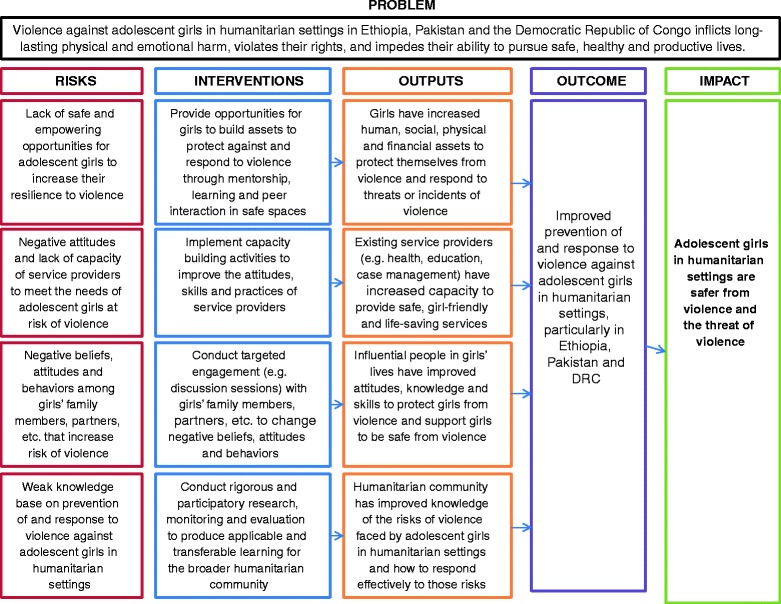
Theory of Change for COMPASS

The theory of change for the COMPASS program is presented in Fig. 1. The core component of the program is to provide opportunities for girls to build assets to protect against and respond to violence and establish a foundation for a healthy transition to adulthood through mentorship, learning, and peer interaction in safe spaces. A specific activity through which this may be achieved is through the implementation of structured, facilitated sessions that focus on topics, tailored for girls, such as self-confidence, building friendships, communication, problem solving, puberty and reproduction as well gender based violence and creating healthy relationships. All of the above are aimed at helping girls prepare for making decisions about adult relationships, including their ability to negotiate sex, and understand their self-worth. In addition, adolescents are asked to create plans to minimize risk of entering relationships where girls may experience abuse, violence, or exploitation. This curriculum has been adapted for age groups in the study and settings, and will be delivered by young female mentors (from their late teens to 30 years old) trained by IRC COMPASS program staff. Over the course of 10–12 months, activities will be delivered in safe spaces for girls: specially constructed *tukuls* (i.e., traditional huts) in Ethiopian refugee camps for only women and girls, and dedicated spaces in community based women’s organizations in DRC. These safe spaces were developed specifically for girls through several community and caregiver consultations.

Caregivers will also participate in monthly discussion groups which seek to increase knowledge of the needs of adolescent girls. The goals are to ultimately shift gender-inequitable attitudes about girls in the home and increase support to keep girls safe within and outside of the home. This content is delivered by IRC program staff. The incremental effectiveness of this caregiver component is being tested in DRC, while in Ethiopia we are evaluating the overall COMPASS program effectiveness.

In addition, COMPASS will engage health service providers and case managers to train workers on Clinical Care for Sexual Assault Survivors [28] and Caring for Child Survivors Guidelines [29] in order to improve the overall quality of services for adolescent girl survivors of violence in their communities. Additional activities will be undertaken to engage communities to support girls as well.

### Randomization, sample size, and power analysis

Based on the assumptions of 80 % power to detect a Cohen’s effect size of d = 0.3 between treatment versus wait-list groups (α < 0.05 statistical significance level, a design effect of 2.0), a sample size of 704 girls is required in each country. After accounting for an expected attrition rate of 25 % in DRC, the final sample size required at least 880 girls and a total of 886 girls were surveyed at baseline. Primary caregivers of selected girls in DRC were also asked to participate in the study (n = 777 surveyed at baseline). In Ethiopia, given movement concerns within the refugee camps and higher likelihood of loss to follow-up and potential refusals at baseline, the attrition rate was inflated and a total of 919 girls were surveyed at baseline.

Groups are comprised of 20–25 girls each, based on geographic proximity or language. Groups of girls were divided into younger groups and older groups, given age-tailored content of programming. In DRC, 35 girl groups across 14 villages in South Kivu were divided into two age strata: 10–12 years of age and 13–14 years of age. In Ethiopia, 47 groups across three refugee camps were divided into age strata; group formation also accounted for school scheduling in the camps. Groups were first stratified by age level (13–14 years and 15–19 years) and then randomized to either the intervention or wait-list arm following the baseline survey. If sisters or girls who identified the same caregiver or reside in the same house were participating in the program, girls were assigned to the same group if they were in the same age category. If they were in separate age groups, the groups were paired together and randomized to the same study arm. Randomization was completed using the statistical software R version 3.2.1.

### Study population, recruitment, and retention

IRC program staff introduced the COMPASS program to the communities, and interested girls and their caregivers were invited to participate in the program. For example, in Ethiopia, outreach efforts included social worker house visits and ongoing activities at IRC Women and Girls Wellness Centers, which provide basic socioeconomic empowerment programming for women and girls in the camps and psychosocial services for survivors. Upon completion of program registration forms, girls (and their caregivers in DRC) were invited to participate in the studies. Eligibility criteria for the program and study varied by age and language based on contextual tailoring of adolescent girl programming across country sites. Details are provided in Table 2. In all sites, potential participants were excluded if they had cognitive impairment. Caregivers and girls were selected to participate in qualitative research activities based on purposive selection to generate a diversity of languages, ages, and schooling statuses.

**Table 2:** Eligibility criteria for COMPASS girl research participants

	DRC	Ethiopia	Pakistan
Age	10-14 years	13-19 years	12-19 years
Language	Swahili	Engesena Quickly^a^	Pashtu
	Mashi	Funj/Berta	
		Maban	
		Regarig	

^a^Can also be termed Ingessana Kulag

Throughout the delivery of COMPASS, girls and their caregivers will be tracked by program mentors to improve and record retention through attendance records. Primary concerns for loss to follow-up for this study includes displacement, lack of buy-in from parents which could result in girls not being allowed to participate, and early marriage. Specifically, the latter is a concern because when girls marry, they may drop out of programming to undertake additional household duties. If a girl becomes married within the duration of the program, she will still be encouraged to participate by the mentors. In addition, if a girl has not attended programming, mentors will directly follow-up with the girl and caregiver and support her attendance. Reasons for non-attendance will be continually assessed by program and research teams; program delivery timing or other barriers to attendance will be addressed as possible. If there are multiple girls that miss more than three sessions within one group, IRC staff will engage directly with the mentor to support that mentor to improve retention of girls in her group.

Further, in Ethiopia, additional steps are planned to retain and track girls in the wait-list group, while minimizing risk of contamination. This tracking is also needed to determine whether girls have moved from the camps during the study period. These steps include checking in with girls during standard school-supply distributions approximately six months into the study and an additional check in with girls one month before endline data collection.

### Ethics

All study procedures were approved by the Columbia University Institutional Review Board (IRB) and by in-country local bodies (Ministry of Health, Gender, Family, and Humanitarian Affairs in South Kivu, DRC and Administration for Refugee and Returnee Affairs in Ethiopia). The study protocol for Ethiopia was also approved by the International Rescue Committee internal review board. All caregivers were asked to provide informed consent for the girls’ participation in the study if the girl was under 18 years old. Subsequently, girls were asked to assent for their participation in the study. In DRC, informed consent was read to potential participants through trained enumerators and written consent was given. Caregivers were also asked to provide written informed consent for their own participation in the quantitative survey and qualitative research activities. In Ethiopia, informed consent was administered via audio recordings because the languages selected for the study are non-written; thus, informed consent forms were verbally translated into the appropriate languages and recorded. All potential participants listened to the same audio recording to ensure consistency in the informed consenting process and provided verbal consent. Data collection staff were trained and available to respond to any questions on the consenting process. Since languages were non-written, only verbal consent/assent was required for participants in Ethiopia.

Given the highly sensitive nature of conducting research on the topic of violence against girls, all research staff completed training on the importance of ensuring confidentiality, privacy, and basic concepts of gender-based violence in accordance with the World Health Organization guidelines on violence against women research [30] and protocols for reporting breaches were developed. All enumerators were asked to complete confidentiality agreements. At the end of the interview, all participants were given information about where and how to receive additional services regarding violence and psychosocial support and referrals were made to service providers as needed.

### Quantitative assessment and analytic plan

The primary outcome measure for the study is change in sexual violence. Other secondary outcomes include physical/emotional abuse, healthy relationships, and self-esteem, for example. Hypothesized directions of changes, primary and secondary outcomes, and measures are found in Table 3. Scales were tested through cognitive interviewing techniques prior to the baseline and adapted as needed in both countries. All study tools were translated into local languages and back-translated into English.

**Table 3:** Primary and secondary outcomes and hypothesized direction of change

	Hypothesized direction of change	Source of measure
Girl Primary Outcomes		
Past-Year Sexual Violence	Decrease	Adapted from CDC Violence Against Children Survey [39]
Girl Secondary Outcomes (Slight variations by country)		
Past-Year Positive Interpersonal Relationships	Increase	Adapted from CDC Violence Against Children Survey [39] & Sisters of Success Liberia survey [40]
Past-Year Early Marriage	Decrease	Adapted from CDC Violence Against Children Survey [39]
Past-Year Physical Violence & Emotional and Verbal Abuse	Decrease	Adapted from IPSCAN Child Abuse Screening Tools [41]
Self-Esteem	Increase	Rosenberg Self-Esteem Scale [42]
Gender Equitable Norms	Increase	Gender Relationship Scale [43]
Hope and Future Orientation	Increase	Children’s Hope Scale [44]
Accepting Attitudes towards domestic violence	Decrease	Demographic and Health Surveys (DHS) Domestic Violence Module [45]
Caregiver Outcomes (DRC Only)		
Parental Acceptance	Increase	Parental Acceptance-Rejection Questionnaire (Parent Short Form) [46]
Accepting Attitudes of Negative Discipline	Decrease	Adapted from Child Protection Knowledge, Attitudes, and Practices (KAP) in Liberia Survey [47]
Gender Equitable Norms	Increase	Gender Norms and Attitudes Scale [43]

In DRC, Audio Computer Administered Self Interviews (ACASI) software was used for sensitive violence questions and Computer-Administered Personal Interviews (CAPI) was used for non-sensitive questions for girls and for the full caregiver survey. Only the older groups of girls (aged 13–14) were asked violence and sexual health modules given ethical concerns.

In Ethiopia, ACASI was used for the entire survey due to challenges of hiring female enumerators who can both speak the local non-written languages selected for the study (Engsena Quickly, Funj/Berta, Maban, or Regarig) and read and write in Amharic, Arabic or English. ACASI was selected as a data collection method as it has been shown to increase reporting of sensitive behaviors [31] in addition to its ability to overcome the language and literacy challenges found among refugee camps in Western Ethiopia. Colors and images for response options were piloted and varied by country in the final ACASI programming.

Descriptive statistics and tests of variables of interest, including chi-square tests and t-tests, will be constructed. Generalized linear mixed models, also known as hierarchal/multilevel mixed models, will be used to assess whether girls in the intervention arm report changes in outcomes of interest compared to girls in the intervention arm, accounting for multiple levels of clustering in the data (time, family, group, village/refugee camp zone). Intention-to-treat analyses and per protocol analyses, based on attendance records, will be generated.

### Qualitative assessment and analytic plan

At baseline, methods of qualitative data collection included focus group discussions with caregivers and social mapping exercises with girls in both countries. In-depth interviews with girls and caregivers were also collected at baseline in DRC only. Similar activities will be conducted at endline. Focus group guides with caregivers sought to identify community gender norms related to girls and key challenges girls may face throughout periods of transitioning to adulthood in their communities, including challenges related to violence. Social mapping activities with girls involved asking girls to draw a map of their community and to note and discuss whether places were safe or unsafe. In-depth interviews with caregivers and girls sought to identify challenges girls face and how members of the family or community can support girls.

In DRC, qualitative data collectors were trained to conduct the group activities and record hand-written focus group or mapping notes in either Swahili or Mashi. In-depth interviews with girls and caregivers were audio recorded, transcribed, and then translated into English. In Ethiopia, Ethiopian qualitative data collectors facilitated the group activities in English with assistance from female Berta/Funj or Regarig translators. Only these two languages were selected for the qualitative research in Ethiopia due to challenges locating available female translators who could also speak either English or Amharic to communicate with the qualitative facilitators. All hand-written notes were recorded in English and subsequently transcribed. A combination of inductive content analysis and grounded theory will be utilized for data analysis to generate connections and themes across qualitative data.

### Monitoring and process evaluation

Three main components comprise the process evaluation for the COMPASS program: (1) attendance records; (2) safe space quality assessments; and (3) service provider quality criteria checklists. All tools have been adapted as needed across countries.

First, attendance of girls and caregivers will be systematically tracked in program activities to understand adherence rates and identify any barriers to participation or potential issues with mentors, and follow up with non-attending girls as appropriate.

Second, the quality of safe spaces and the quality of programming delivered within the spaces will be assessed through three separate tools. Activities to assess the levels of learning of curriculum topics will be conducted at least twice during the curriculum cycle, which focus on girls’ perceptions of favourite topics, usefulness of different content modules, and any unintended consequences of the programming. A similar method will be used to capture perceptions and knowledge gained from caregivers participating in the program. In addition, mentors will be supported to share their perceptions of girls’ reactions to the programming and the appropriateness of delivery (i.e., space, timing, methods, etc.), through supervision meetings. IRC staff will also conduct observations of safe space sessions to provide overall feedback to mentors and to fidelity of session delivery to the curriculum guidelines.

The third component for the process evaluation will focus on assessment of service provider quality through training feedback forms, observation reports of case managers and health workers, child client satisfaction surveys, and analysis of GBV Information Management System data to capture the number of survivors receiving services from the IRC or other providers in the study areas.

Monitoring data will be used as a mechanism to explain the impact evaluation results, guide per-protocol analyses as findings emerge, and to provide data for other program logical framework indicators that fall outside the scope of the evaluation studies.

## Discussion

The COMPASS evaluation will yield important insights into potential strategies to improve the safety of adolescent girls in humanitarian settings by addressing their specific needs and challenges. Importantly, these research studies within COMPASS will assist in unpacking the utility of different approaches, such as caregiver discussion groups to support the empowerment of adolescent girls and reduce girls’ risk of violence. These studies will also demonstrate the potential effectiveness of safe spaces and the programming within safe spaces to improve outcomes specifically related to adolescent girls’ safety, above and beyond children’s protection needs previously examined in safe space research [32, 33]. The diversity of study sites (i.e., communities in DRC, refugee camps in Ethiopia, and camps or host communitiess in Pakistan) will also provide valuable learning on how programming should be tailored to different phases of humanitarian emergencies or displacement. However, given the need to adapt measures to the different cultural contexts, the ability to generalize broadly across populations may be limited.

Throughout the inception period and launch of the two randomized controlled trials, a number of challenges emerged and protocols have been adapted accordingly, as detailed below. First, while the original intention of the evaluation was to recruit only unmarried girls, as a key secondary outcome of the study is early marriage, the inclusion criteria had to be loosened. Though all of the girls initially reported themselves to be unmarried in program enrollment forms, which served as the basis for participant recruitment into the studies, they later reported that they were married when asked through ACASI in both DRC and Ethiopia. In Ethiopia, it is thought that this discrepancy was due to the illegal nature of early marriages in Ethiopia [34] and thus, participants did not report their marriages to program staff prior to launching the survey. As found in other research, child brides may be less able to benefit from violence prevention programs as compared to women married as adults, given the additional social vulnerabilities they may face [35]. Therefore, additional programmatic attention must be paid to ensure that married girls are not only retained in the program, but also receive supportive messages and tailored content.

Second, the diversity of languages was also challenging in the Ethiopian refugee camps. A language assessment was conducted by the program team to determine which languages girls spoke, the level of fluency in each language they were reported to speak, as well as the total number of girls who spoke a language with sufficient fluency that they could understand ACASI in order to ensure sample size requirements could be met. Based on these assessments, as well as a mapping of available and qualified female research assistants or translators that were able to communicate in camp languages, the final four languages were selected for the quantitative survey and two languages for the qualitative activities. While the process behind selecting languages was methodically carried out, some refugee leaders reported the final selection had potential to exacerbate community tensions as only certain languages were selected for inclusion into the research study. Leaders preferred all surveys to have been completed through informal Arabic, which most girls could understand to some degree, but not to the fluency that the survey questions required which would have ultimately limited the reliability and validity of the survey. Importantly, girls speaking any languages will be invited to participate in the program, regardless of whether they are part of the research sample.

Other biases have been considered in the study design as well. Primarily, contamination is of concern as intervention groups and wait-listed groups may interact with each other which may minimize our ability to detect changes between groups. Due to sample size requirements for cluster-randomized controlled trials, groups were randomized within communities in DRC and within small geographical areas in refugee camps in Ethiopia. Potential contamination will be assessed during the endline data collection. Social desirability bias may also emerge if respondents in the intervention arm report lower levels of violence, for example, to please the interviewer. However, the use of ACASI may minimize this bias as interviewers will not be aware of the girls’ responses. In addition, another concern is that humanitarian settings are comprised of highly mobile populations, such that girls and caregivers may move out of the study area during the period of program delivery. Attendance will be tracked and intention-to-treat and per-protocol analyses will be undertaken to assess how this attendance may impact the effectiveness of COMPASS. Finally, behavior change to reduce violence against women and girls may require long term investments and our ability to see changes in the limited follow-up period may be minimized. Therefore, examination of secondary outcomes, such as gender attitudes and norms will also be critical to interpretation of the effectiveness of COMPASS as well as the potential salience of different pathways to change. As seen in other community-based trials to reduce intimate partner violence, consistency, trends, and alignment with hypothesized directions of change of both primary and secondary outcomes will be examined to provide insight into potential effectiveness [19, 20, 36, 37].

Despite these challenges, the studies have been strengthened by intensive engagement with in-country program teams as they contextualize the program and research within the political and cultural environments. Rigorous attention to such contextualization by country teams has proven critical for successful engagement with community leaders, adaptation of measures used in the survey, as well as general recruitment of girls and caregivers into the study. Nonetheless, such contextualization, piloting, and attention to translations and language issues extended the research program’s inception and initial planned period of approximately three months to almost one year. This led to a substantial delay in launching the COMPASS programming activities and strained relationships with communities as the period between program registration and launch of activities was lengthened.

In conclusion, while undertaking these studies has proven challenging, they provide further evidence that rigorous impact research can be undertaken in humanitarian emergencies on sensitive topics [20, 38], particularly through strong engagement with local team members and communities. Findings will provide much needed evidence for the humanitarian community, donors, and governments to guide actions and investments in promoting the safety and wellbeing of adolescent girls affected by armed conflict and crises.

### Trial status

At the time of initial manuscript submission, baseline surveys in Ethiopia and DRC have been completed. The study is ongoing.

## Abbreviations

ACASI: Audio-Computer Assisted Self-Interview
CAPI: Computer-Administered Personal Interview
COMPASS: Creating Opportunities through Mentorship, Parental involvement, and Safe Spaces
DRC: Democratic Republic of Congo
IRC: International Rescue Committee
